# A plant-based chemical genomics screen for the identification of flowering inducers

**DOI:** 10.1186/s13007-017-0230-2

**Published:** 2017-10-03

**Authors:** Martijn Fiers, Jorin Hoogenboom, Alice Brunazzi, Tom Wennekes, Gerco C. Angenent, Richard G. H. Immink

**Affiliations:** 1 Bioscience, Wageningen University and Research, 6700 AP Wageningen, The Netherlands; 2 Laboratory of Molecular Biology, Wageningen University and Research, 6708 PB Wageningen, The Netherlands; 3 Laboratory of Organic Chemistry, Wageningen University and Research, 6708 WE Wageningen, The Netherlands; 4 Department of Chemical Biology and Drug Discovery, Utrecht Institute for Pharmaceutical Sciences and Bijvoet Center for Biomolecular Research, Utrecht University, 3584 CH Utrecht, The Netherlands

**Keywords:** Chemical genomics, Flowering, Arabidopsis, *APETALA1*, Luciferase, Salicylic acid

## Abstract

**Background:**

Floral timing is a carefully regulated process, in which the plant determines the optimal moment to switch from the vegetative to reproductive phase. While there are numerous genes known that control flowering time, little information is available on chemical compounds that are able to influence this process. We aimed to discover novel compounds that are able to induce flowering in the model plant Arabidopsis. For this purpose we developed a plant-based screening platform that can be used in a chemical genomics study.

**Results:**

Here we describe the set-up of the screening platform and various issues and pitfalls that need to be addressed in order to perform a chemical genomics screening on Arabidopsis plantlets. We describe the choice for a molecular marker, in combination with a sensitive reporter that’s active in plants and is sufficiently sensitive for detection. In this particular screen, the firefly Luciferase marker was used, fused to the regulatory sequences of the floral meristem identity gene *APETALA1 (AP1)*, which is an early marker for flowering. Using this screening platform almost 9000 compounds were screened, in triplicate, in 96-well plates at a concentration of 25 µM. One of the identified potential flowering inducing compounds was studied in more detail and named Flowering1 (F1). F1 turned out to be an analogue of the plant hormone Salicylic acid (SA) and appeared to be more potent than SA in the induction of flowering. The effect could be confirmed by watering Arabidopsis plants with SA or F1, in which F1 gave a significant reduction in time to flowering in comparison to SA treatment or the control.

**Conclusions:**

In this study a chemical genomics screening platform was developed to discover compounds that can induce flowering in Arabidopsis. This platform was used successfully, to identify a compound that can speed-up flowering in Arabidopsis.

**Electronic supplementary material:**

The online version of this article (doi:10.1186/s13007-017-0230-2) contains supplementary material, which is available to authorized users.

## Background

Because plants are sessile, they have to carefully monitor their growing conditions to determine the optimal moment of flowering. This floral timing is a precisely regulated process, in which the plant determines the best moment to switch from the vegetative to the reproductive phase and to produce its offspring in the form of seed. The discovery of the elusive flowering hormone Flowering Locus T (FT), which is a major inducer of flowering in many plant species, was a major breakthrough in flowering research [1, 2]. This ‘florigen’ is produced in the leaves and transported to the shoot apical meristem (SAM), where it induces the onset to flowering [1]. Beside this mobile flowering inducer and the phytohormone Gibberellin [3], there are very few proteins or compounds known that are able to affect flowering time upon exogenous application. There are a few examples of compounds that can influence flowering, like the FN analogues in Lemna (duckweed), or anilide and benzamide derivatives in Aspargus [4, 5]. Nevertheless, screening a large collection of compounds in a high throughput manner, aiming to identify flowering inducing compounds, has not been reported yet. We aimed to develop a high throughput chemical genomics screening platform, suitable for the identification of novel flowering inducing compounds, which potentially can be implemented to obtain flowering on demand. Controlling the transition from vegetative growth to flowering is important for plant breeders and growers. Additionally, identified flowering time modifying chemicals can be used as research tool to get a better understanding of the complex regulatory network underlying this biological process in different plant species [6].

While there are numerous genes involved in flowering time regulation, there are very few chemical compounds known that influence this process. The advantage of using compounds over a genetics approach is their transient mode-of-action, circumventing potential problems with lethality and providing possibilities to get around genetic redundancy, in case a compound targets a set of redundantly acting proteins [7, 8]. For this purpose, we set out to screen a large collection of compounds in the model plant *Arabidopsis thaliana*. Before such a chemical genomics screen could be performed, we first had to establish the parameters and conditions for the screen. Media and growth conditions for growing Arabidopsis in 96-well plates were optimized, and a suitable flowering marker gene was cloned and fused to the sensitive luciferase reporter.

This study describes the development of a chemical genomics screening platform for the identification of flowering inducing compounds, and discusses the various parameters that were taken into account. Using this chemical genomics platform, a compound named ‘Flowering1’ (F1) was identified that was able to speed-up flowering in Arabidopsis in the plate assay, but also upon watering of soil-grown plants with this compound. F1 turned out to be a novel analogue of the plant hormone Salicylic acid (SA). SA was already implicated to be involved in the induction of flowering [9, 10], but F1 turned out to be more potent in the induction of this important developmental switch.

## Methods

### DNA vector construction and transformation

For the reporter construct, we amplified a 5.4 kB genomic fragment containing the *APETALA1 (AP1)* gene (AT1G69120) from *Arabidopsis thaliana* Col-0 (lacking the stop codon and including a 1.8 Kb *AP1* promoter) using primers GGGGACAAGTTTGTACAAAAAAGCAGGCTCCGCTT-ACTACTTTTGCTCATGATCTC and GGGGACCACTTTGTACAAGAAAGCTGGGTTTGCGGCGAA-GCAGCCAAGGTTGCAG, comprising gateway sites, which was previously shown to drive the expected *AP1* expression pattern [11]. This genomic fragment was recombined with a BP reaction into vector pDonR207 (Invitrogen). This donor vector was used in an LR reaction to recombine the genomic *AP1* fragment into the destination vector *pGREEN GW*-*FLuc*, resulting in *pAP1::AP1*-*FLUC* in a *pGREEN* vector backbone. This vector was transformed to *Agrobacterium tumefaciens* C58, containing the helper plasmid pSOUP. *A.thaliana* Col-0 was transformed using the flower dip method [12]. The transgenic plants were tested for the correct expression of the *AP1* transgene and for single locus mendelian inheritance (3:1 ratio). Subsequently, a homozygous *pAP1::AP1*-*FLUC* progeny plant was selected. Seeds of this homozygous transgenic Arabidopsis line were used in all chemical genomics screens. In order to minimize variation, one large seed lot was produced that was subdivided into smaller portions and stored at − 20 °C to ensure a constant seed quality and subsequent uniform germination during screening.

### Chemical genomics screen

The DIVERSet-CL chemical library (Chembridge) is a 10,000 compound library obtained from Chembridge as 10 mM stocks dissolved in DMSO in 96-well plates. The Library of AcTive Compounds on Arabidopsis (LATCA, http://cutlerlab.blogspot.com) is comprising~ 3700 compounds, and this library was dissolved in DMSO as a 2.5 mM stock in 96-well plates. In both libraries the first and last row of each plate are controls and contain only the solvent DMSO (0.25 and 1% for the Chembridge and LATCA library, respectively), resulting in 16 controls/plate. The compounds and controls were diluted in sterile water to a final concentration of 1 mM. Using these diluted stocks, 3.75 µl was pipetted in triplicate to three new white flat bottom 96-well plates with a translucent lid (Greiner). To these compounds 150 µl of ½ Murashige and Skoog (MS, Duchefa, NL) medium with 0.5% sucrose and 0.5 g/L MES (pH 5.8) was added, which results in a final compound concentration of 25 µM.

For the chemical genomics screen *Arabidopsis thaliana* Col-0 seeds were gas sterilized for 2 h using 100 ml of bleach combined with 3 ml of HCl in a closed container. Seeds were dispersed (one seed/well), using an Arabidopsis seed loader (vp-scientific). The plates with the seeds were stratified for 3 days at 4 °C, after which the plates were transferred to a growth chamber at 20 °C with either 8 or 16 h of light/day for a period of 12 days.

**Fig. 1:**
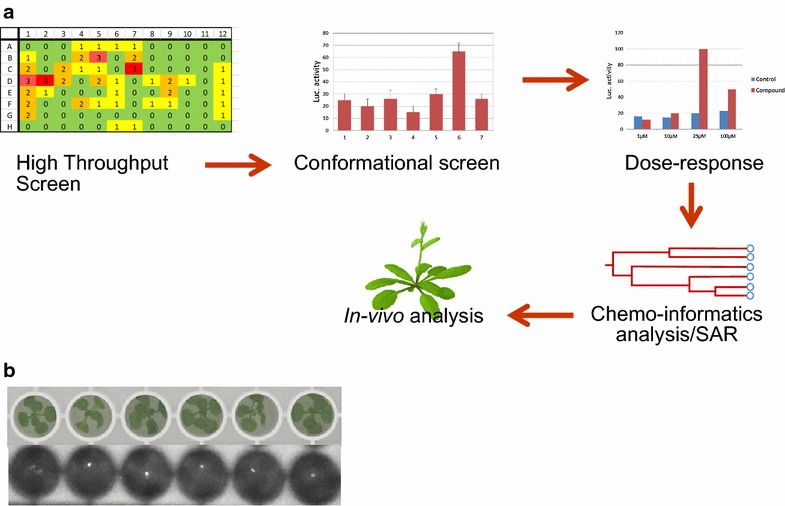
Chemical genomics screening set-up for flowering. **a** Schematic representation of the chemical genomics workflow implemented in the screen for potential flowering inducing compounds. **b** Example of *pAP1::AP1*-*LUC* plants in a 96-well plate. Plants were grown for 16 days at 20 °C, after which they were sprayed with luciferin and measured. Note that the majority of plants is switched and showing luciferase signal in the central shoot apical meristem

The firefly luciferase substrate d-luciferin (P-salt, Goldbio, U.S.A.) was dissolved in water at a final concentration of 1 mM with 0.05% of Tween 80, after which the solution was filter sterilized. After 12 days of growth, the plants were sprayed with luciferin and incubated in the dark for 1 h, after which the plates were analysed on a Glomax luminometer (Promega), with 2 s integration time per well. Data from the luminometer was analysed in excel. If the average luciferase signal of the compound (from the three replicates) was more than the average signal of the DMSO control samples plus two times the standard deviation ($\bar{X}$Fluc compound > $\bar{X}$Fluc DMSO + 2.SD), a compound was considered a putative initial hit. For qualitative analysis (Fig. 1b), plants were sprayed with luciferin, incubated in the dark for 1 h, after which the plants were analysed with a G-box (Syngene) with 40 min integration time.

### Luciferase interaction test

To determine if a compound may affect directly the activity of luciferase, we used the protocol as described for AID 588342 (https://pubchem.ncbi.nlm.nih.gov/bioassay/588342). We dispersed 100 µl of reaction buffer per well, containing 50 mM Tris acetate, pH 7.5, 10 mM Mg acetate, 0.01% Tween-20, 0.05% BSA, 10 µM d-luciferin (P-salt, Goldbio, U.S.A.), and 10 µM ATP in a white 96-well plate (Greiner). One µl of each individual compound, dissolved in DMSO, was added with a final concentration range of 0.01–25 µM, with DMSO as control treatment. Finally 12.5 µl of 40 nM firefly luciferase (Promega) in a 500 mM Tris-acetate buffer was added in each well. Luciferase activity was measured on a Glomax luminometer (Promega), with 2 s integration time per well.

Ataluren 3-(5(2-Fluorphenyl)-1,2,4-oxadiazol-3-yl)benzoic acid (Fluorochem, U.K.) was used a positive control [13].

### GUS analysis

GUS histochemical staining was performed in a staining solution containing 50 mM sodium phosphate buffer, pH 7.0, 1 mM EDTA, 0.5 mg/ml 5-brom-4-chloro-3-indolyl ß-DGLcUa (X-Gluc, Duchefa), 0.4% Triton X-100, 2.5 mM potassium ferrocyanide and 2.5 mM potassium ferricyanide. The leaf tissue was incubated in the staining solution at 37 °C for 24 h in the dark. After the staining the chlorophyll in the tissue was removed by an 1 h incubation in 96% ethanol followed by several washes with 70% ethanol until the tissue was translucent.

### qRT-PCR analysis

Total RNA from 12 day old seedlings was isolated with the Invitrap Plant Spin RNA Mini Kit (Invitek) and treated with DNAseI (Invitrogen). First-strand cDNA was synthesized from 1 µg of DNAseI treated total RNA using the Iscript mix from Biorad in a 20 μl reaction. The cDNA was diluted 20-fold and 4 µl was used for each qPCR reaction. qPCR reactions were run on the BioRAD myIQ system using SYBRgreen (Biorad) in a final volume of 20 µl [PCR program; 3 min. 95 °C, 40 × (15 s 95 °C, 1 min 60 °C)]. The sequences of the forward and reverse qPCR primers used to quantify *AP1* are TGCCTCTGGTTTCTCTCCAAAAGC and CGCTATGAGAGGTACTCTTACGCCG, respectively.

## Results and discussion

### Considerations and initial set-up of the screening platform

In this study, we aimed to develop a chemical genomics screening platform for the identification of compounds that can induce flowering in Arabidopsis. However, the majority of discussed points and considerations are also relevant for chemical genomics screens in other plant species and for other traits.

One of the first choices that has to be made in a chemical-genomics screening deals with the biological material that will be used. This can either be a specific protein (in vitro), isolated plant cells, a plant organ, or even a whole seedling or plant. The choice depends on the goal of the experiment and research question to be answered. Flowering is a complex process regulated by various environmental and endogenous signals and in Arabidopsis hundreds of genes have been identified involved in this process [14]. Because we didn’t aim to focus on a specific flowering time pathway, we decided to screen at whole plant level.

A well-known problem in chemical genomics is the transition from in vitro to in vivo, meaning that identified compounds in a screen at protein or single cell level are not active or toxic at plant level, and this is circumvented in our screen by screening at whole plant level.

The model plant *Arabidopsis thaliana* is an ideal plant for the use in a chemical genomics screen, because it is a small-sized and fast cycling plant, in which the flowering pathway is well studied. Despite these optimal characteristics, a visually flowering Arabidopsis plant will not fit in a well of a 96-well plate, which is commonly used for large-scale chemical screens. Therefore, we were in need of a molecular reporter that marks the transition to flowering earlier than flowering can be observed visually. For this purpose we selected the floral meristem identity gene *AP1*, because it’s not expressed during the vegetative stage of development and is specifically activated in the newly formed floral meristems directly after the transition from vegetative to reproductive development and 2–3 weeks before flowering can be observed visually [15, 16]. Initially *AP1* is expressed in only a few cells in the centre of the floral meristem, which increases over time when the flower meristem is developing further, making it a quantitative marker of flowering time in Arabidopsis [17].

Because of the low *AP1* expression immediately after the switch to flowering it’s essential to use a reporter which is sensitive enough to be detected when expressed in only a few cells. Furthermore, this reporter should be non-destructive to permit sequential measurements. β-glucuronidase (GUS) is a sensitive marker, but the various fast and sensitive detection assays for this enzyme are destructive. Green Fluorescent Protein (GFP) might be detected when expressed in a few cells, but suffers from high background signals due to auto-fluorescence when applied in aboveground green plant tissues. Therefore, we selected Firefly (*Photinus pyralis*) LUCIFERASE (FLuc) as reporter. FLuc is highly sensitive, has a large dynamic range, and can be measured rapidly in plants [18]. Arabidopsis is commonly grown in translucent 96-well plates but these kind of plates are not suitable for screening with the luciferase reporter, which has light as an output and would cause cross illumination in these plates. For this reason the plants were grown and analysed in non-translucent white 96-well plates, with a single seedling per well.

Arabidopsis can be grown under a wide variety of environmental conditions, but we selected a flowering-inducing long day growth regime (16 h light, 8 h dark) to reduce the time needed for our screenings and to overcome problems with plants becoming too large without flowering. We also performed a pilot experiment, in which we grew Arabidopsis in 96-well plates under short day conditions (8 h light, 16 h dark), but this resulted in stressed plants, showing hyperhydricity and no signal of flowering.

Using the above set-up, we determined when Arabidopsis switched to flowering in 96-well plates under long day conditions, based on luciferase expression of the *pAP1::AP1*-*FLuc* transgene. We measured luciferase activity at different time points after germination. At the first time point, 10 days after germination, none of the plants had switched, while after 12 days around 10–20% of the plants showed a luciferase signal and hence, were switched from vegetative to reproductive development. After 16 days, almost all plants had made the transition to flowering. Based on these observations, the fact that plants which are too young and still in their juvenile phase are not responsive to flowering inducing cues, and that we wanted a sensitive screening platform, we decided to screen after 12 days of growth in the presence of the compound in the medium [19, 20]. Due to this short time to grow the plants, small changes in vitality and grow speed may have a clear influence on the flowering time and hence, outcome of the screening. Therefore, particular emphasis has to be given to seed quality and uniformity in such a chemical genomics screening set-up.

In order to deal with the biological variation, we decided to screen each compound in triplicate. Three replicate plates were generated from each master plate with compound stocks. Note that this setup results in a similar arrangement of compounds over all three plates, providing no extra information on positional effects. Compounds were considered positive in the initial screen if the average luciferase activity of the compound (over the three independent plates) was higher than the average luciferase activity of the plants grown on the DMSO containing control medium in the same plates, including two times the standard deviation ($\bar{X}$Fluc compound > $\bar{X}$Fluc DMSO + 2.SD).

The choice of the compound library is a very important part of a chemical genomics screen given the large variety of available chemical libraries and the bias some of these libraries possess in the molecular structure of their compounds. We chose a combination of an untargeted, structurally diverse synthetic compound library and a targeted plant specific compound library. This selection was made, because we were not targeting a specific enzyme or protein and aimed to keep the screen as broad as possible (For review see [21, 22]). For the non-targeted approach, we used a synthetic library of 10,000 compounds (DIVERSet-CL). This was combined with the Library of AcTive Compounds on Arabidopsis (LATCA), comprising ~ 3700 compounds. The LATCA library is a mixed library consisting of herbicides, common inhibitors, plant hormones, research chemicals and other bioactive compounds which are geared towards a use in plants and for influencing plant-specific biological processes. Ideally, a screen would be performed at multiple concentrations of the compounds, to reduce the possibility that a potential lead compound is missed because it was not tested at its optimal concentration, i.e. a concentration too low for activity or too high, causing toxicity. Furthermore, this would give insight into the dose–response of the compound. However, because of the size of the selected libraries and to keep the screening feasible, we decided to screen at one fixed concentration of 25 µM [23, 24].

### General observations and results of the chemical genomics screen

We screened the complete LATCA-library and half of the DIVERSet-CL library in triplicate, comprising a total of around 8700 compounds, following the experimental flow as described in Fig. 1. The compounds from the different libraries were screened at a concentration of 25 µM with the dissolvent DMSO as a control. The initial screen resulted in a hit rate of 2.1 and 3.1% for the DIVERSet-CL and LATCA library, respectively. The hit rates we observed in our screens are relatively high as compared to other primary chemical genomics screens, which can vary between less than one and up to a few percent [25]. The differences in the initial hit rates between the DIVERSet-CL and LATCA library may be explained in the differences in the composition of the libraries and concentration of the compounds in the original libraries, resulting in adding different amounts of the solvent DMSO for the two libraries. We noticed that luciferase activity, and hence flowering time, was affected slightly by DMSO as a stressing agent (Additional file 1: Fig. S1), making the DMSO controls an essential part of the screen.

We analysed the results of the primary screen for the distribution of the hits over the plate and the average luciferase activity per well (Additional file 1: Fig. S2). We noticed that the hits were not uniformly distributed over the plates with less hits in the middle of the plate, which coincides with an on average lower luciferase signal (Additional file 1: Fig. S2A, B). One possible explanation of this plate effect is the fact that there was condensation of water against the lid in the middle of the plate, especially at the end of the growing period, which resulted in a delay in plant growth and sometimes vitrified plants. This delay in growth could be the reason for the lower number of hits and an on average lower luciferase signal in the middle of the plate.

The leads from the primary screen were re-tested in a conformational screen and positive compounds from this second screen were re-ordered and tested in a dose–response experiment to confirm the initial result and to determine at which concentration the compounds give the strongest effect on flowering time (Fig. 1). Only three compounds remained positive after re-ordering and re-testing, and these appeared to be structurally related compounds. The structural relationship was found using a chemo informatics analysis to detect potential overlap in the structures of the positive compounds (https://pubchem.ncbi.nlm.nih.gov; Additional file 1: Fig. S3).

**Fig. 2:**
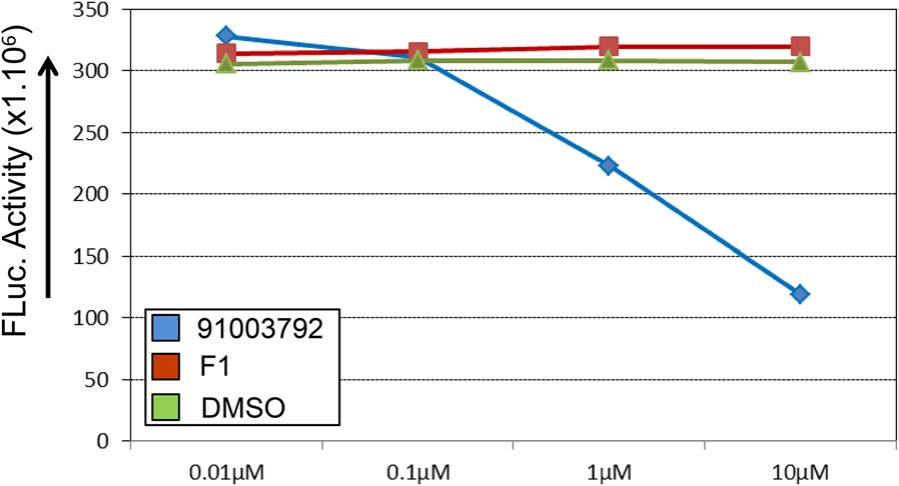
In vitro screening for Luciferase-affecting compounds. Outcome of the analysis of two initial hits and the control DMSO in an in vitro Fluc assay for luciferase inhibition or activation. As shown, compound 91003792 from the DIVERSet-CL library is having a direct effect on the FLuc reporter, while the compound with the highest Luciferase inducing activity in the initial flowering time screening, named F1, has no influence on Fluc activity. The compounds were dissolved in DMSO, which itself has no effect on Luciferase activity

Beside confirming initial leads, we were also cautious for false positives due to an interaction of the compound and the reporter luciferase. Previously, it was found that up to 60% of the identified hits in a given luciferase based screen were actually inhibitors of luciferase and thus false positives or negatives, depending on the reporter set-up and desired trait [26]. To explore this possibility, we decided to test 20 compounds identified as potential flowering inducer in the initial screen (Fig. 1) for their ability to directly affect luciferase activity. It was shown that Fluc-inhibitor-based stabilization can be caused by compounds that bind and stabilize the luciferase protein and as such, extend the half-life of the protein resulting in more Fluc and an increased and potential false positive signal [26]. To identify such potential false positives we used an in vitro assay, in which FLuc was mixed with luciferin and different concentrations of the compounds (Fig. 2). Out of the 20 compounds that were tested for any interaction with Fluc, only two compounds turned out to be positive. Instead of performing this in vitro Luciferase activity assay, putative positive compounds can be re-ordered and re-tested on wild type plants, followed by visual scoring of flowering time. In this way, independent confirmation of a flowering time effect will be obtained and false positives due to an effect on the Luciferase reported can be excluded.

### Towards the identification of a flowering inducing compound

When the initial hits from the LATCA library were analysed to identify clusters of chemically related compounds, there was one cluster that caught our attention, comprising of three compounds. These leads were very similar in structure and were all above the threshold luciferase signal in the conformation screening (Fig. 1; Additional file 1: Figs. S3, S4). Based on the positive effect of these related compounds on flowering, we decided to name the consistently best performing compound ‘Flowering 1’ (F1) and to study it in more detail. Interestingly the three compounds closely resembled the plant hormone Salicylic acid (SA) and while SA itself was present in the LATCA library, it didn’t induce flowering under our initial screening conditions.

**Fig. 3:**
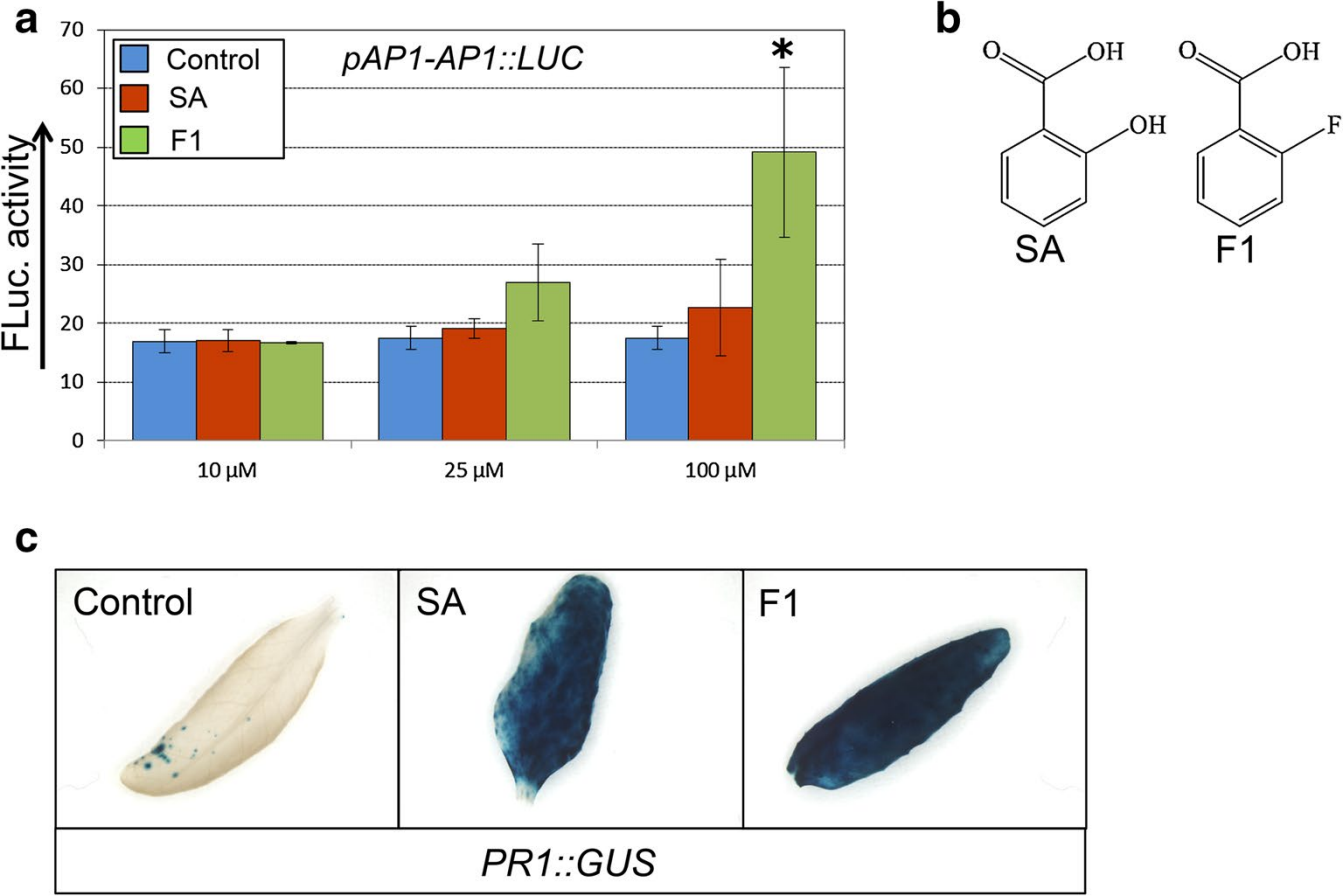
F1 is an flowering inducing compound that is similar to SA. **a** F1 and SA were re-tested at 10, 25 and 100 µM on *pAP1::AP1*-*LUC* plants in 96-well plates, with water as a control. Plants were grown for 12 days before luciferase measurement. Average of five replicates with 24 plants/replicate. Error bars represent SE. Significant differences at p value < 0.05 (Student’s t-test) compared to the control are marked with ‘*’. **b** Structure of SA and F1. **c** GUS assay on leaves from a *pPR1::GUS* transgenic Arabidopsis line incubated for 24 h with 25 µM of F1, SA, or water as a control

Although SA is best known for its function in defence, it recently has been associated with flowering as well [10, 27] and exogenous application of SA has already been shown to induce flowering in Arabidopsis [9]. Therefore, we re-ordered F1 and tested it at different concentrations in comparison to the DMSO control and treatment with SA (Fig. 3a, b). This step was still performed in a 96-well format with *pAP1*::*AP1*-*FLuc* plants, but with a larger number of individuals. The treatment resulted in an increase in luciferase activity with F1 at 25 µM and an even stronger and significant effect at 100 µM. However, SA didn’t significantly induce *AP1* expression under our screening conditions, even not at 100 µM (Fig. 3a).

To determine the specificity of the F1 structure we tested a close analogue of F1, in which the fluoro-substituent is shifted from the ortho- to the para-position (compound F1-4F) and compared this to the outcome for F1 and two of the other SA analogues (compound A and B), which were positive in the initial screen and clustered in the same clade as F1 (Additional file 1: Figs. S3–S5). All the compounds of the initial identified SA-like cluster were positive, as expected, but no induction of *AP1* was found for F1-4F. This observation reveals that there is a strict structure–activity relationship for the ability of F1 to induce flowering (Additional file 1: Fig. S5).

Because F1 strongly resembles SA, and SA is known as a key signalling molecule in the plant pathogen response, we analysed whether F1 may can affect the plant pathogen response as well. For this purpose we used a *PR1::GUS* reporter line, which is commonly used as a marker for the defence pathway and is strongly induced by SA [9, 28]. In leaves, containing the *pPR1::GUS* reporter, *GUS* expression was strongly induced by SA, but also by F1, showing that F1, like SA, is able to induce *PR1* expression (Fig. 3c).

There are many synthetic SA-analogues known that can act as synthetic plant defence elicitors and it’s not known if these SA-analogues can also induce flowering in Arabidopsis in a similar fashion as F1 [29, 30]. One possible explanation of the stronger activity of F1 then SA in the induction of flowering is that F1 cannot be glucosylated by glucosyltransferases as the fluoro-group on the second position of F1 replaces the key hydroxyl group required for the formation of SA 2-O-β-glucoside (SAG). Glucosylation inactivates SA and allows vacuolar storage, resulting in a reduction of the amount of bio-available SA [31]. This was also shown with the immune-priming compound Imprimatin, which inhibits glucosyltransferases and as such elevates endogenous bio-active SA levels [32]. Whether F1 resembles a continuously active non-glycosylated form, and whether other synthetic SA analogues can induce flowering, needs further investigation.

**Fig. 4:**
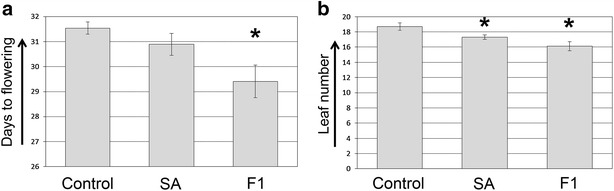
Effect of exogenous application of F1 and SA on flowering time. Wild type Arabidopsis plants were treated daily, from day six after sowing with 100 µM of F1 or SA dissolved in water with 0.01% Tween80. **a** Days to flowering. **b** Total number of rosette leaves at the moment of bolting. Values are the mean of three replicates with 30–40 plants/replicate with SE. Significant differences at p value < 0.05 (Student’s t-test) compared to the control are noted with ‘*’

So far we tested all our compounds for their effect on the induction of flowering on transgenic Arabidopsis plants grown on plant media in 96-well plates. The ultimate test for a putative flowering inducing compound is to water or spray wild type (WT) Arabidopsis plants with the compound of choice and to measure flowering time. To subject our F1 lead compound to this critical test, WT plants were watered and sprayed continuously, from day six after sowing onwards, with either 100 µM F1, 100 µM SA, or water as a control. The number of days to flowering (moment that bolting starts and the inflorescence is just visible) was determined, as well as the number of rosette leaves at this moment. For the flowering time expressed in number of days to bolting, only F1 treated plants displayed a significantly shorter time (p < 0.05) from the control, while for the number of leaves both F1 and SA were significantly different from the control, which concurs with a previously reported study on SA (Fig. 4a, b, [9]). In both days to flowering and leaf number, F1 displayed a stronger phenotype than SA, which is consistent with the results obtained in the 96-well plate-based luciferase assay (Figs. 3a, 4a, b).

### Problems and possible improvements of the Chemical genomics screen

When (Arabidopsis) plants are grown in 96-well plates covered with a lid, it is difficult to obtain equal aeration of all wells in the plate. Consequently, condensation of water against the lid often occurs in the middle of the plate. Amongst others, this condensation leads to variation in growth and development of the plants between wells and plates and might be one of the major causes of the plate effect that was observed for the average luciferase activity and distribution of the initial hits over all the analysed plates (Additional file 1: Fig. S2A, B). One possible solution is to screen in larger wells (24 well plates), in which the plants have more space and will cause less problems with condensation after 2 weeks of growth. The downside of this approach is the increase in the amount of work, the seed loader cannot be used anymore, pipetting will take much more time (from a 96 well library to a 24 well screening plate), and the increased amount of compound that is needed for the screen due to the need for a larger volume of medium per well.

Another problem was the relative high hit rate in our initial screen and the low confirmation rate and general lack of reproducibility. The hit rates in primary chemical genomics screens differs a lot between screens and may vary between less than one and up to a few percent [25]. In this flowering time screen, the hit rate in the initial screen was 2.1 and 3.1%, for the DIVERSet-CL and LATCA libraries, respectively. This is quite high, but can still be dealt with. The main problem in our flowering time screen was the short window for the compounds to act. The first plants already started to flower after 12 days at long day conditions, resulting in a high background, which we tried to deal with by performing the screen in triplicate. Screening at an earlier time point might solve this problem, but is also not ideal because Arabidopsis plants that are too young and still in their juvenile phase and therefore, most likely not competent to flower. Growing at non-inductive short day conditions sounded as an attractive alternative, but unfortunately, these conditions resulted in stressed plants. A combination of using 24 well plates (less condensation and stress) with a reduction of light conditions (12 h of light) might be instrumental in solving at least part of the problems.

## Conclusions

In this paper we describe a plant based chemical genomics screening platform, which was developed to find novel compounds that enhance flowering in Arabidopsis. Various aspects of such a screen were discussed and optimized in such a way that Arabidopsis plants that contain a *pAP1::AP1*-*FLuc* reporter construct, can be screened in a 96-well format. This set-up allows medium to high throughput screening for compounds that are potentially able to induce flowering.

The novel set-up resulted in the identification of a new SA-analogue that we call Flowering1 (F1). Beside the induction of flowering in tissue culture plates, this compound was also able to induce flowering in wild type Arabidopsis plants grown in the growth chamber on rock wool. Taken together, we developed a chemical genomics approach to identify compounds that can induce flowering in a plant-based in plate screen, using the *AP1* marker combined with luciferase as reporter.

## Additional file

**Additional file 1:** **Fig. S1.** Effect of DMSO on flowering time of Arabidopsis in 96 well plates. Effect of DMSO on *pAP1::AP1*-*LUC*. Plants were grown for 12 days in 96-well plates with different concentrations of DMSO, after which the luciferase activity was measured. **Fig. S2.** Distribution of initial hits from the Chembridge library. **A** Distribution of the average luciferase activity from 56 96-well plates of the initial screen from the Chembridge library. **B** Distribution of the number of initial hits from all screened 96-well plates from the Chembridge library. Columns 1 and 12 are controls containing DMSO. Note that hits were more often found at the borders of the plate pointing towards a position effect due to the screening conditions and set-up. The wells are colour coded based on the average luciferase activity (A), or number of hits (B). **Fig. S3.** Structure clustering of the SA-analogues with SA. Positive SA-analogues (F1, A, and B) from the screen were clustered together with SA in Pubchem (pubchem.ncbi.nlm.nih.gov). **Fig. S4.** Results from a selection of initial screening plates that contained F1 and its derivatives. The screen was performed in triplicate with the DMSO controls in the first and last column of each plate. The Fluc values for F1 and its analogues are colour coded. **Fig. S5.** Result of a Structure Activity Relationship (SAR) analysis for F1. Two positive compounds from the initial screen similar in structure to F1 (Compound A and B), and one analogue of F1 (Compound F1-4F) were retested for the induction of *AP1* expression. Compounds were tested at 25 µM against p*AP1::AP1*-*LUC* plants in 96-well plates with water as a control (compounds were dissolved in water). Plants were grown for 12 days before luciferase measurement. Error bars represent SE of six replicates with 16 plants/replicate.
