# Supplementary material for: A plant-based chemical genomics screen for the identification of flowering inducers
Source: Plant Methods. 2017 Oct 3;13:78. doi: 10.1186/s13007-017-0230-2 (PMC5627458; doi:10.1186/s13007-017-0230-2)
Supplement: Supplementary file 1 — Additional file 1: Fig. S1. Effect of DMSO on flowering time of Arabidopsis in 96 well plates. Effect of DMSO on pAP1::AP1-LUC. Plants were grown for 12 days in 96-well plates with different concentrations of DMSO, after which the luciferase activity was measured. Fig. S2. Distribution of initial hits from the Chembridge library. A Distribution of the average luciferase activity from 56 96-well plates of the initial screen from the Chembridge library. B Distribution of the number of initial hits from all screened 96-well plates from the Chembridge library. Columns 1 and 12 are controls containing DMSO. Note that hits were more often found at the borders of the plate pointing towards a position effect due to the screening conditions and set-up. The wells are colour coded based on the average luciferase activity (A), or number of hits (B). Fig. S3. Structure clustering of the SA-analogues with SA. Positive SA-analogues (F1, A, and B) from the screen were clustered together with SA in Pubchem (pubchem.ncbi.nlm.nih.gov). Fig. S4. Results from a selection of initial screening plates that contained F1 and its derivatives. The screen was performed in triplicate with the DMSO controls in the first and last column of each plate. The Fluc values for F1 and its analogues are colour coded. Fig. S5. Result of a Structure Activity Relationship (SAR) analysis for F1. Two positive compounds from the initial screen similar in structure to F1 (Compound A and B), and one analogue of F1 (Compound F1-4F) were retested for the induction of AP1 expression. Compounds were tested at 25 µM against pAP1::AP1-LUC plants in 96-well plates with water as a control (compounds were dissolved in water). Plants were grown for 12 days before luciferase measurement. Error bars represent SE of six replicates with 16 plants/replicate. [file 13007_2017_230_MOESM1_ESM.pptx]

## Slide 1
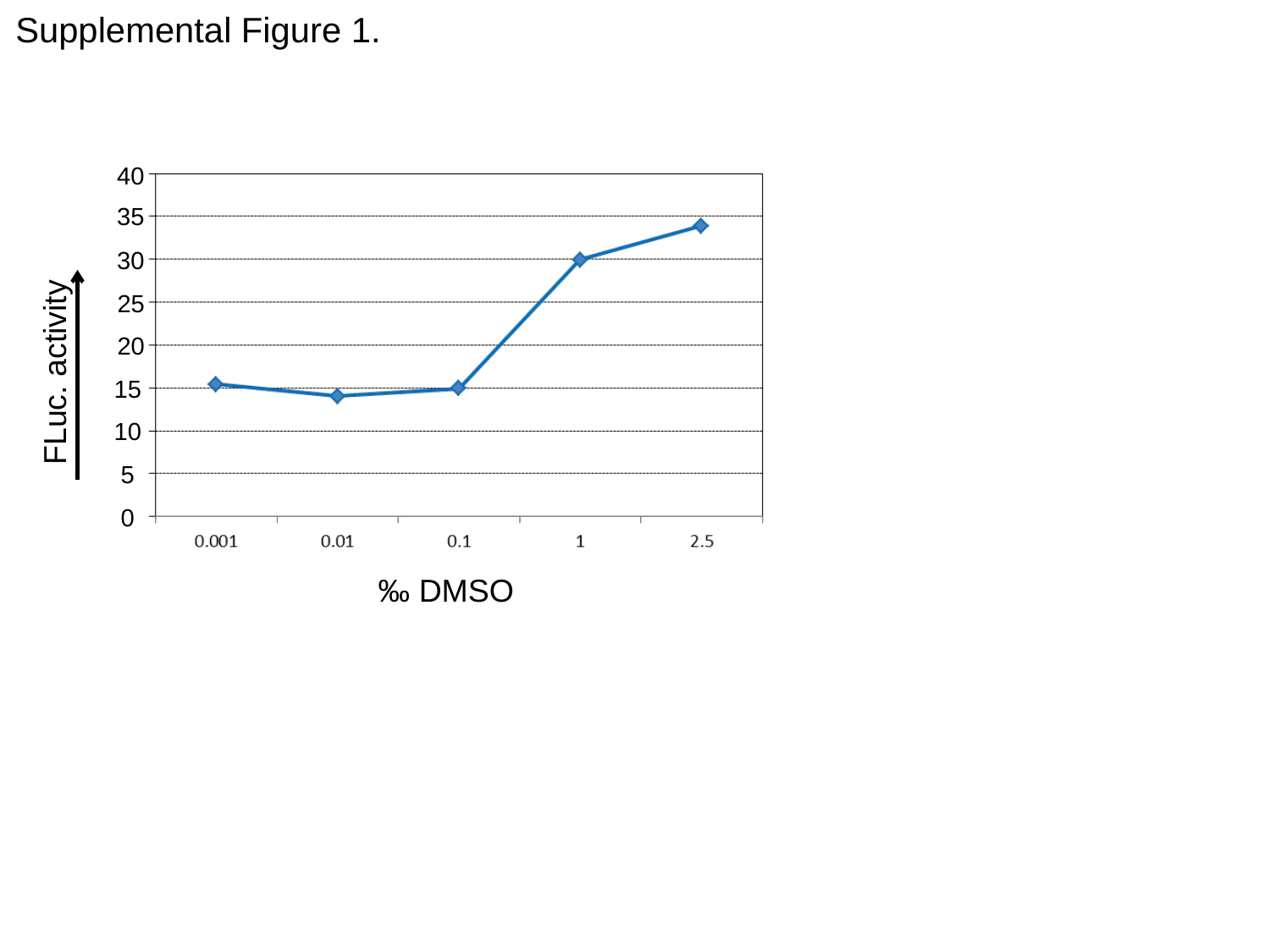

Supplemental Figure 1.
40
35
30
25
20
FLuc. activity
15
10
5
0
‰ DMSO

## Slide 2
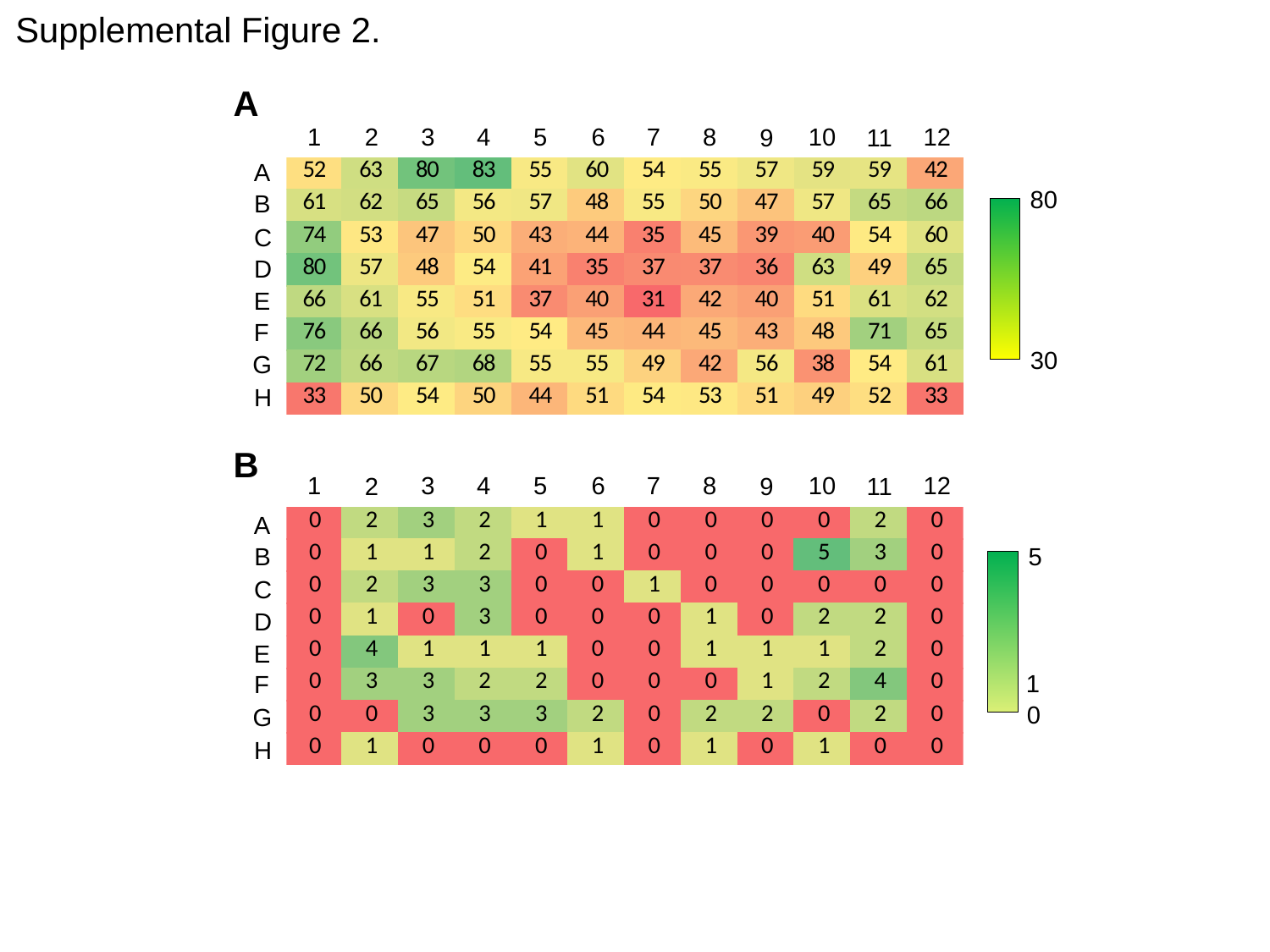

Supplemental Figure 2.
A
3
5
8
10
7
6
4
12
1
2
9
11
A
80
B
C
D
E
F
30
G
H
B
3
5
8
10
7
6
4
12
1
2
9
11
A
B
5
C
D
E
1
F
0
G
H

## Slide 3
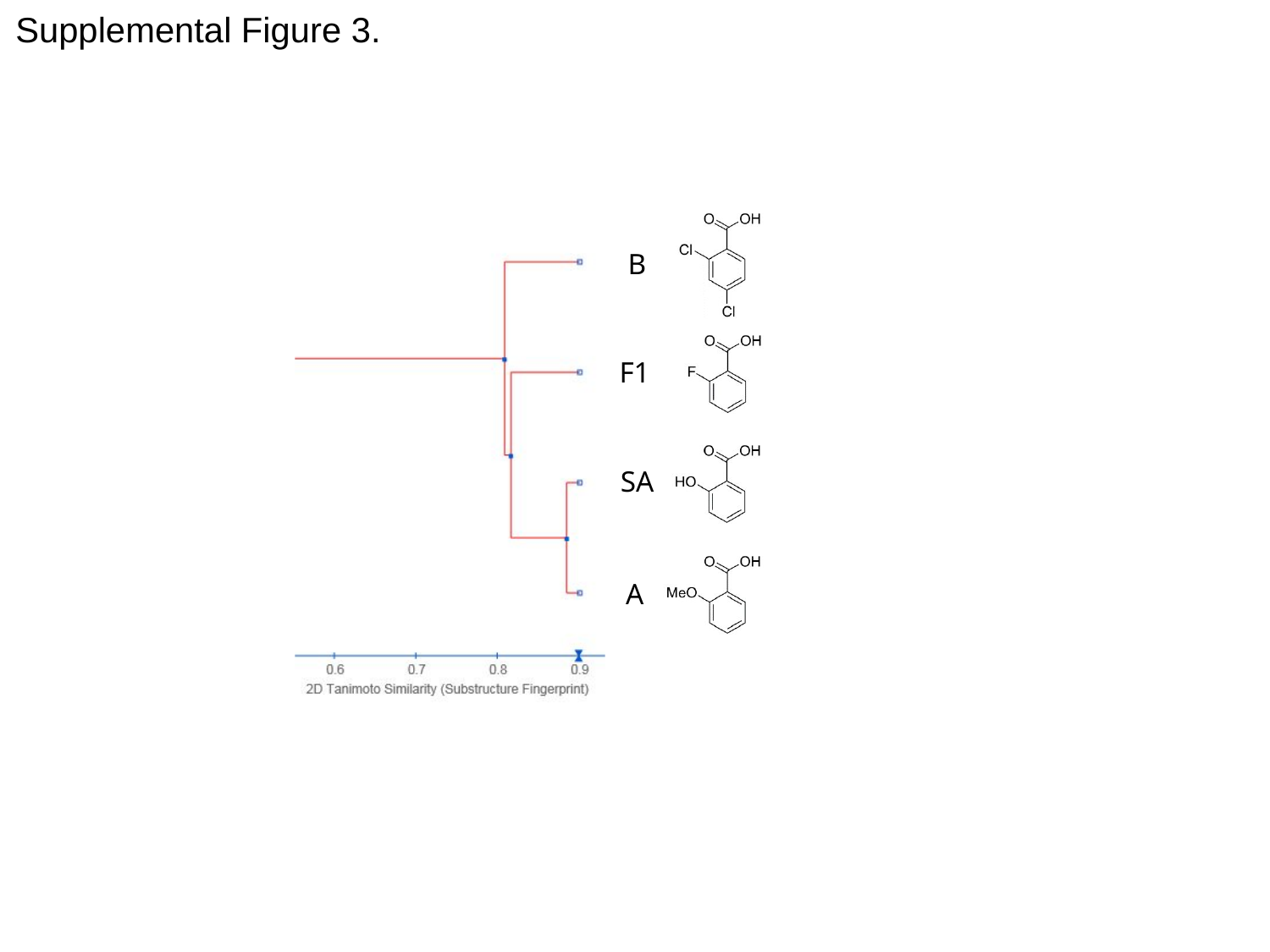

Supplemental Figure 3.
B
F1
SA
A

## Slide 4
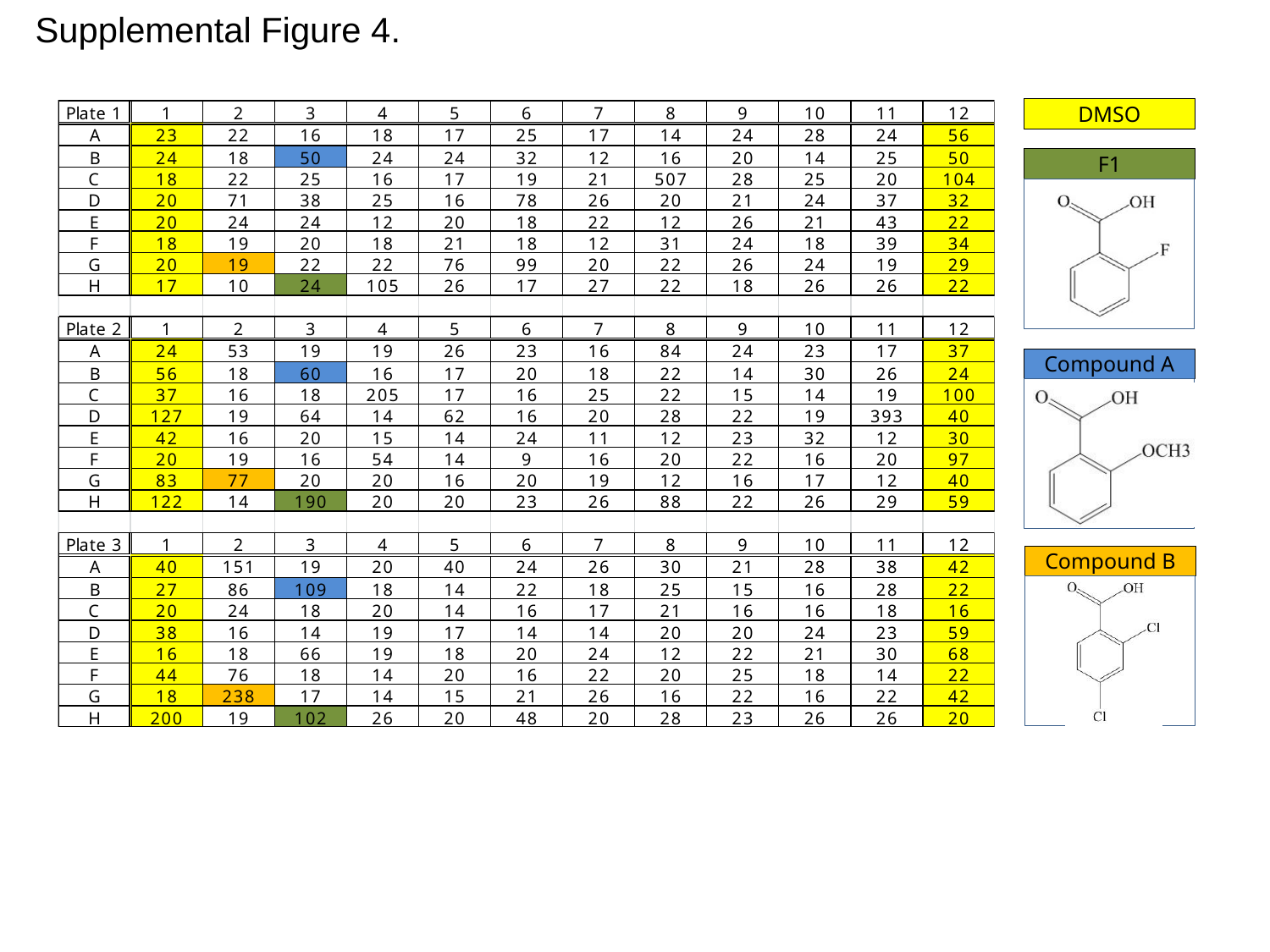

Supplemental Figure 4.
DMSO
F1
Compound A
Compound B

## Slide 5
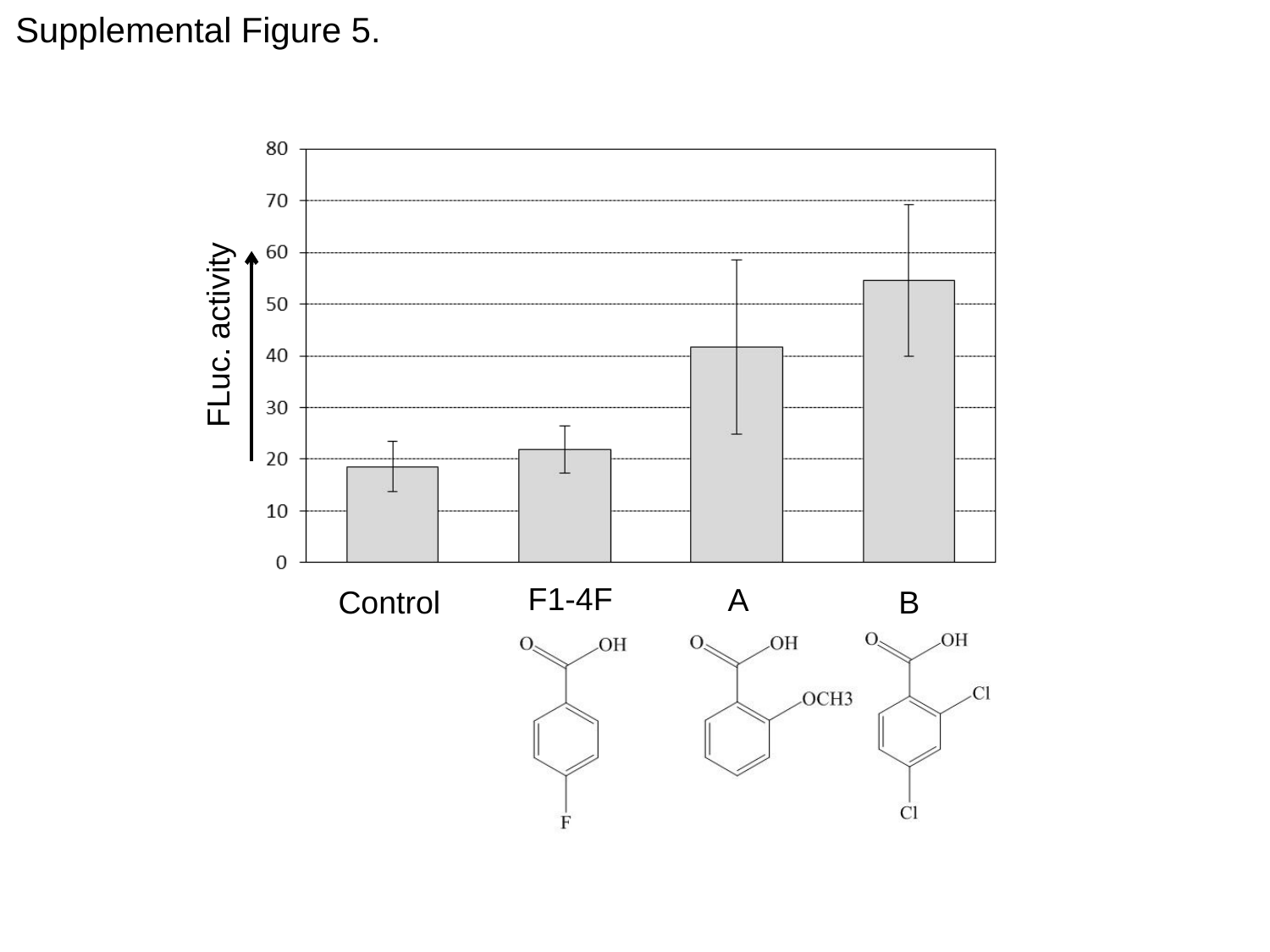

Supplemental Figure 5.
FLuc. activity
F1-4F
A
Control
B
